# Effectiveness of the Gratuité user fee exemption policy on utilization and outcomes of maternal, newborn and child health services in conflict-affected districts of Burkina Faso from 2013 to 2018: a pre-post analysis

**DOI:** 10.1186/s13031-023-00530-z

**Published:** 2023-07-06

**Authors:** Marie-Jeanne Offosse, Cephas Avoka, Pierre Yameogo, Astrid Raissa Manli, Aude Goumbri, Ejemai Eboreime, Matt Boxshall, Aduragbemi Banke-Thomas

**Affiliations:** 1 ThinkWell, 11 B.P. 1255 CMS 11 Ouagadougou - Quartier Ouaga 2000, près de la fondation Kimi, à 500 du boulevard Muammar Kadaffi, Ouagadougou, Burkina Faso; 2 Department of Infectious Disease Epidemiology, Faculty of Epidemiology and Population Health, London School of Hygiene and Tropical Medicine, London, UK; 3 Ministry of Health, Ouagadougou, Burkina Faso; 4 Department of Psychiatry, Faculty of Medicine and Dentistry, University of Alberta, Edmonton, Canada

**Keywords:** User fee, Health policy, Universal health coverage, Conflict, Effectiveness, Burkina Faso

## Abstract

**Background:**

Evidence on effectiveness of user fee exemption policies targeting maternal, newborn, and child health (MNCH) services is limited for conflict-affected settings. In Burkina Faso, a country that has had its fair share of conflicts, user fee exemption policies have been piloted since 2008 and implemented along with a national government-led user fee reduction policy (‘SONU’: Soins Obstétricaux et Néonataux d'Urgence). In 2016, the government transitioned the entire country to a user fee exemption policy known as Gratuité. Our study objective was to assess the effect of the policy on the utilization and outcomes of MNCH services in conflict-affected districts of Burkina Faso.

**Methods:**

We conducted a quasi-experimental study comparing four conflict-affected districts which had the user fee exemption pilot along with SONU before transitioning to Gratuité (comparator) with four other districts with similar characteristics, which had only SONU before transitioning (intervention). A difference-in-difference approach was initiated using data from 42 months before and 30 months after implementation. Specifically, we compared utilization rates for MNCH services, including antenatal care (ANC), facility delivery, postnatal care (PNC) and consultation for malaria. We reported the coefficient, including a 95% confidence interval (CI), *p* value, and the parallel trends test.

**Results:**

Gratuité led to significant increases in rates of 6th day PNC visits for women (Coeff 0.15; 95% CI 0.01–0.29), new consultations in children < 1 year (Coeff 1.80; 95% CI 1.13–2.47, *p* < 0.001), new consultations in children 1–4 years (Coeff 0.81; 95% CI 0.50–1.13, *p* = 0.001), and uncomplicated malaria cases treated in children < 5 years (Coeff 0.59; 95% CI 0.44–0.73, *p* < 0.001). Other service utilization indicators investigated, including ANC1 and ANC5+ rates, did not show any statistically significant positive upward trend. Also, the rates of facility delivery, 6th hour and 6th week postnatal visits were found to have increased more in intervention areas compared to control areas, but these were not statistically significant.

**Conclusions:**

Our study shows that, even in conflict-affected areas, the Gratuité policy significantly influences MNCH service utilization. There is a strong case for continued funding of the user fee exemption policy to ensure that gains are not reversed, especially if the conflict ceases to abate.

## Background

There is strong evidence in the literature showing that conflict affects maternal, newborn and child health (MNCH) service utilization [1–5]. One country currently tackling significant insecurity threats which has impacted all spheres of life including health is Burkina Faso—especially in the northern part of the country [6]. This crisis has led to over 1·9 million people (8.1% of the country’s total population) being forcibly displaced from their homes and over 600 health facilities (42% of the total) having reduced services including 211 health facilities which are completely closed because of direct attacks by unidentified armed groups, as of February 2023 [7]. There have been emerging discussions on strategies needed to protect and maintain provision of essential health services during conflict, more so in a time when the global attention is on meeting the Sustainable Development Goal (SDG) 3 [8]. However, another critical piece of the jigsaw is maintaining utilization of services. One approach that has long been utilized in many settings including conflict-affected ones is user fee exemption, which experts deemed to be important for African countries if universal health coverage (UHC) is to be realized [9].

In Burkina Faso, the first pilot project for a user fee exemption scheme started in the late 2000s [10], followed by another between 2008 and 2015, implemented in partnership with non-governmental organizations (NGOs), and financed by the European Civil Protection and Humanitarian Aid Operations (ECHO) in specific districts of the country. These districts where the NGO-led user fee exemption pilot was implemented were Dori and Sebba (in the Sahel region), Tougan (Boucle du Mouhoun region), and Séguénéga (Nord region). During the pilot, NGOs subsidized 100% of the direct payment for the care received by pregnant women and children under-5 years in all public health facilities. In these districts, the user fee exemption pilot ran parallel to the national user fee reduction policy for delivery and emergency obstetric and newborn care (locally referred to as ‘SONU’: Soins Obstétricaux et Néonataux d'Urgence) put in place by the government to subsidize 80% of the cost of accessing maternal health services [11, 12].

Following its adoption by the Council of Ministers of Burkina Faso on 2nd March 2016, the government rolled out the Gratuité user fee exemption policy nationally on 1st June 2016. Since then, the policy has been implemented in all public and some private health facilities across the country. As per design, public facilities provide a defined package of MNCH services free at point of use to service users. Instead of charging out-of-pocket payments, equivalent fee-for-service payments are made to facilities by the central government. To date, between 60 and 80% of the funds are earmarked for drugs, and facilities can use the remainder for services, such as consultations. The scheme is managed by the government and verification is contracted out to NGOs. The exemptions from the direct payment for essential health care services offered due to the policy align with the country’s commitment to universal health coverage (UHC) and is expected to contribute to attaining the SDGs. The policy's long-term vision is to significantly reduce avoidable deaths among children aged 0–5 years and women [13, 14].

Indeed, available evidence shows that reducing or eliminating user fees leads to increased utilization and improved health outcomes, especially for children and women in low- and middle-income countries (LMICs), including Burkina Faso [15–18]. However, evidence on effectiveness of user fee exemption policies is varied, mostly based on uncontrolled studies, and limited for conflict-affected settings. The Gratuité policy now in its sixth year of implementation offers a good substrate for analysis, which will contribute immensely to this knowledge gap. Our objective in this study was to assess the effect of the policy on the utilization and outcomes of MNCH services in conflict-affected areas of the country. The key hypothesis underpinning this objective was that outcomes in the post-implementation period would be better than in the pre-implementation period for districts that have not had a user fee exemption policy applied before the Gratuité policy.

## Methods

### Study design

We conducted a before-and-after/quasi-experimental study to assess the effectiveness of the Gratuité user fee exemption policy on utilization and outcomes of MNCH services in conflict-affected regions of Burkina Faso, comparing 42 months prior to the implementation of Gratuité and 30 months post-implementation. This design is one of the most frequently used methods in impact evaluation studies where treatment assignment is non-random [19, 20].

### Study setting

Burkina Faso is a land-locked West African country with an estimated population of about 22 million in 2021. The country is made up of 13 regions and 63 health districts each with one district or regional hospital. Six of its 13 regions including Boucle du Mouhoun, Centre-Nord, Est, Nord, Hauts-Bassins, and the Sahel have been particularly affected by the conflict [6]. As per the country’s Demographic Health Survey conducted in 2010, before the conflict, antenatal care coverage at least four times was between 13 and 30% in the conflict-affected regions, lower than in the other regions which ranged from 35 to 52%. Similarly, skilled birth attendance was lower in all but one of the conflict-regions (> 75%) compared to the non-conflict ones (mostly < 70% and Sahel region at 36%) [21]. Conflict-affected regions such as Boucle du Mouhoun (13%), Sahel (14%), and Est (16%) had some of the lowest proportion of children < 5 years who had a fever and had received anti-malarial treatment. Though estimates from non-conflict regions such as Centre-Est were higher (32%), some other regions also had low proportions e.g., Sud-Ouest (15%) and Cascades (14%) [21].

**Fig. 1:**
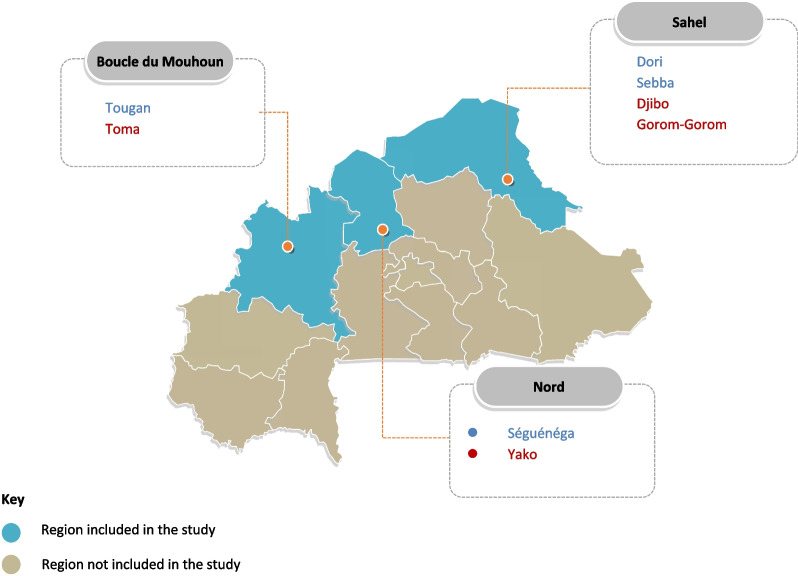
Map of Burkina Faso showing the study regions, highlighting the intervention (in red) and comparator (in blue) districts

Our evaluation was conducted in selected districts within three conflict-affected regions of Burkina Faso—Boucle du Mouhoun, Nord, and Sahel. Since the Gratuité policy was effectively rolled out nationally within a month [13], it was difficult to establish a counterfactual for a large-scale nationwide or regional impact evaluation. A similar concern was highlighted by other authors while evaluating the preceding user fee reduction policy—SONU [11]. With the consideration of the policy implementation approach in mind, for this evaluation, using information from the MoH, districts in the country which had the NGO-supported user fee exemption pilot along with SONU before transitioning to the Gratuité scheme (SONU + NGO-supported exemption pilot → Gratuité [exposed pre-Gratuité]) were identified. This exposed pre-Gratuité group (which was considered the comparator group) was constituted by the following districts (regions in bracket): Dori, Sebba (Sahel), Tougan (Boucle du Mouhoun), and Séguénéga (Nord). These four districts were paired with districts with similar characteristics, which had SONU only and no NGO-supported exemption pilot before transitioning to the Gratuité scheme (SONU only → Gratuité [non-exposed pre-Gratuité group]). The non-exposed pre-Gratuite districts (which were considered the intervention group) selected for this evaluation were Djibo, Gorom-Gorom (Sahel), Toma (Boucle du Mouhoun) and Yako (Nord) (Fig. 1). All selected districts had not been exposed to the performance-based financing (PBF) scheme, which was also being implemented in the country during the study period [22].

### Data collection

We collected data on monthly indicators of MNCH service utilization in the 42 months preceding the launch of the Gratuité policy (January 2013–June 2016) and 30 months afterwards (July 2016–December 2018) for selected districts in conflict-affected regions. Data for both periods (pre- and post-policy) were collected from the Health Management Information System (HMIS). This included data on completeness of antenatal care (ANC) utilization in line with national guidelines, deliveries including normal and cesarean births, postnatal care (PNC) consultations, child clinic visitations, cases of uncomplicated and severe malaria in children < 5 years, neonatal and maternal deaths. For the post-policy period, we used the e-Gratuité database, which collects data from the public health system to quality check data collected from the HMIS. Also, data on denominators were also collected for the various variables. These denominators included the population of children under 1 year, the population of children 1 to 4 years, women of childbearing age, the number of expected pregnancies, and the number of live births (Table 1).

**Table 1:** Outcome measures and anticipated effects

Outcome	Outcome measure	Expected change in the intervention district	Expected change in the comparison district
*For pregnant women*			
ANC 1 coverage	Number of ANC1 women seen/Number of expected pregnancies	Increase	Remain same
ANC 5+ coverage	Number of ANC5+ women seen/Number of expected pregnancies	Increase	Remain same
Women seen in first trimester	Number of women seen in 1st trimester/number of expected pregnancies	Increase	Remain same
Delivery rate	Number of women who delivered in a health facility/number of expected pregnancies	Increase	Remain same
Caesarean section rates*	Number of caesarean sections/number of live births	-	-
Women seen in postnatal consultations in the 6th hour	Number of women seen in postnatal consultations/number of live births	Increase	Remain same
Women seen in postnatal consultations on the 6th day	Number of women seen in postnatal consultations/number of live births	Increase	Remain same
Women seen in postnatal consultations in the 6th week	Number of women seen in postnatal consultations/number of live births	Increase	Remain same
Intra-facility maternal mortality ratio (per 100,000 live births)	Number of intra-facility maternal deaths/number of intra-facility live births	Decrease	Remain same
Cases of malaria in pregnancy seen in health facilities	Number of pregnant women seen with malaria seen in health facilities/number of women of childbearing age	Increase	Remain same
*For children*			
New consultations for children under 1 year	Number of new consultations for children < 1 year/total population of children < 1 year	Increase	Remain same
New consultations for children 1–4 years	Number of new consultations for children 1–4 years /total population of children 1–4 years	Increase	Remain same
Intra-facility early neonatal mortality rate (per 1000 live births)	Number of intra-facility deaths of children 0–6 days/number of intra-facility live births	Decrease	Remain same
Cases of uncomplicated malaria in children < 5 years in health facilities	Number of children < 5 years with uncomplicated malaria in health facilities/total population of children < 5 years	Increase	Remain same
Cases of severe malaria in children < 5 years in health facilities	Number of children < 5 years with severe malaria in health facilities/total population of children < 5 years	Increase	Remain same

*Difficult to predict expected change

### Data analysis

We used a difference-in-difference approach, commonly used to evaluate the impact of health policies [23]. With the difference-in-differences approach, estimating an unbiased effect of a policy requires the assumption that post-intervention trends of the comparator group provide a comparator for ‘what would have happened in the absence of the exposure’ [24]. Parallel pre-intervention trends (between the exposed pre-Gratuité and non-exposed pre-Gratuité groups) of the outcome are considered necessary and sufficient for this assumption [23].

To apply the difference-in-difference approach, we calculated the difference in the outcome (Y) between the before and after situations for the exposed pre-Gratuité group (B − A). We also calculated the difference in the outcome (Y) between the before and after situations for the non-exposed pre-Gratuité group (D − C). Then we calculated the difference between the difference in outcomes for the exposed pre-Gratuité group (B − A) and the difference for the non-exposed pre-Gratuité group (D − C), or difference-in-differences (DID) = (B − A) − (D − C). This approach helped us establish whether the Gratuité policy made a difference at all in terms of access to care, comparing districts with and without the user fee exemption policy in the pre-Gratuité period. The paired districts were Dori versus Djibo, Sebba versus Gorom-Gorom, Tougan versus Toma, and Séguénéga versus Yako. Specifically, in testing effectiveness of the policy, we compared the proportion of women and children who used MNCH services (utilization) or experienced a care outcome (mortality) across the two periods and in both groups to make our inference. As monthly data of the denominators were unavailable, monthly statistics were obtained by dividing annual data by 12. The coefficient, including a 95% confidence interval (CI), *p* value, and the parallel trends test results, were reported. Also, the graphical diagnostics of parallel trends for each outcome showing the observed means of the outcome measures between intervention and comparator districts are compared to a linear trends model of these observed means. The late neonatal mortality rate was excluded from the analysis due to insufficient data. Additionally, the Dori–Djibo pair was excluded from the analysis of caesarean section rates due to the absence of an operating theatre in Dori.

STATA SE 17.0 (StataCorp, College Station, Texas, United States) was used for the analysis.

## Results

In summary, the policy led to a significant increase in rates of 6th day postnatal visits for women, new consultations in children under 1-year, new consultations in children 1 to 4 years, and rates of uncomplicated malaria cases seen in children under 5 years, as shown by their *p* values less than or equal to 0.05 (Table 2).

**Table 2:** Modelled parallel trends between the intervention and control areas

Outcome	Coefficient*	95% CI	*p* value	Parallel trends test^$^
ANC 1 rate	0.029	− 0.012, 0.071	0.141	0.185
ANC 5+ rate	− 0.016	− 0.101, 0.068	0.659	0.497
Rates of women seen in the first trimester of pregnancy	0.047	− 0.037, 0.132	0.227	0.162
Delivery rate	0.015	− 0.025, 0.054	0.408	0.146
Caesarean section rate *(excluding the Dori–Djibo pair)*	− 0.0004	− 0.012, 0.011	0.936	n/a
Rates of postnatal visits in the 6th hour	0.015	− 0.122, 0.151	0.809	0.960
Rates of postnatal visits on the 6th day	0.151	0.010, 0.291	0.039	0.029
Rates of postnatal visits in the 6th week	0.089	− 0.026, 0.205	0.111	0.119
Rates of malaria cases in pregnancy	0.001	− 0.004, 0.006	0.586	0.262
Facility maternal mortality ratio (per 100,000 live births)	− 46.164	− 136.724, 44.396	0.267	n/a
Facility early neonatal mortality rate (per 1000 live births)	− 0.290	− 1.670, 1.091	0.635	n/a
Rates of new consultations in children under 1 year	1.799	1.128, 2.469	< 0.001	0.153
Rates of new consultations in children under 1–4 years	0.813	0.497, 1.129	0.001	0.231
Rates of uncomplicated malaria cases in children under 5 years	0.588	0.443, 0.734	< 0.001	0.002
Rates of severe malaria cases in children under 5 years	− 0.002	− 0.016, 0.013	0.788	0.166

*Coefficient for Average Treatment Effect on the Treated (ATET), which is adjusted for group effects and time effects. The coefficient value signifies how much the mean of the dependent variable changes given a one-unit shift in the independent variable while holding other variables in the model constant. A positive coefficient indicates that as the value of the independent variable increases, the mean of the dependent variable also tends to increase. A negative coefficient suggests that the dependent variable tends to decrease as the independent variable increases. The confidence interval shows the range of possible values that explain the relationship between rates comparing intervention and control areas

^$^Parallel-trends test (pre-treatment period). H_0_: Linear trends are parallel

n/a: Could not be estimated due to insufficient data

**Fig. 2:**
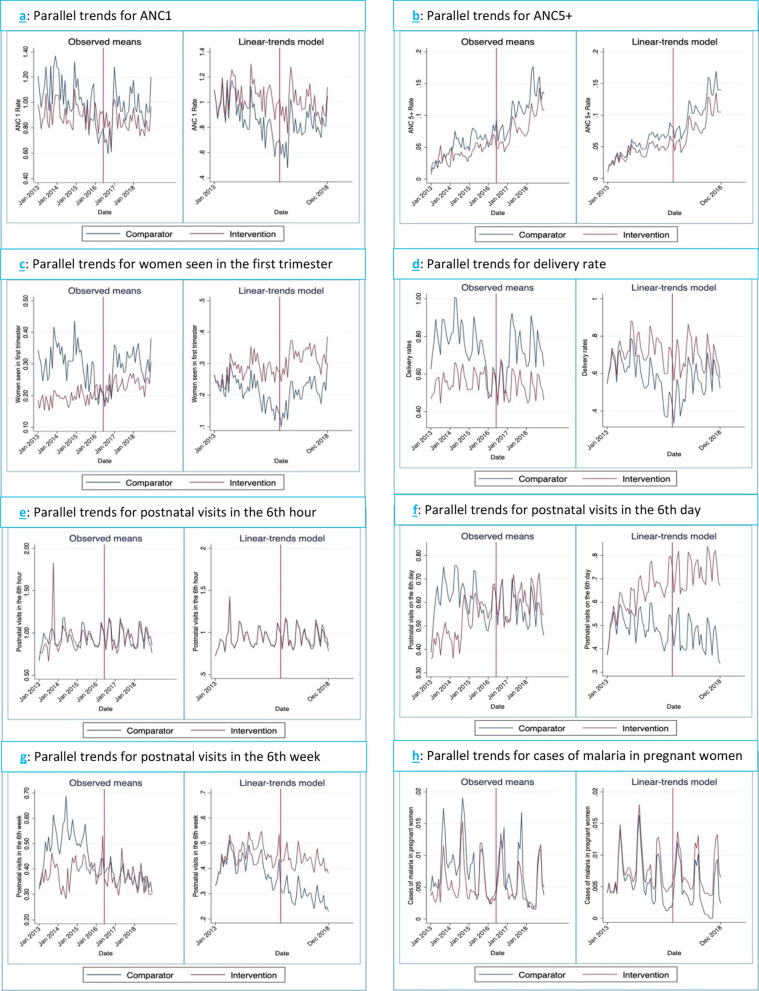
Graphical diagnostics of parallel trends for maternal health indicators

Figure 2; Panel a–h shows the graphical diagnostics of parallel trends for the outcomes investigated in this study relating to the mothers. The expected change in each outcome and the graphical depiction of their diverging trends are observed in the linear trends model after the start of the intervention (depicted by the red vertical line). These lines graphically depict the modelled parallel trends provided in Table 2. The Gratuité intervention led to an increase in rates of women who were seen in health facilities on 6th day postnatal visits (Coeff 0.15; 95% CI 0.01–0.29) in the intervention districts compared to comparison areas and above what would have been observed in the intervention areas if the intervention had not been implemented (counterfactual). However, there was insufficient evidence to assume a parallel trend existed between intervention and comparison districts prior to the intervention (*p* = 0.029) (Fig. 2; Panel f).

Other outcomes that were investigated to determine the impact of the intervention, including ANC1 rates, ANC5+ rates, and rates of women seen in the first trimester of pregnancy, did not show any statistically significant positive upward trend as was expected. In addition, the rates of delivery, 6th hour and 6th week postnatal visits were found to have increased in intervention areas compared to control areas, although these were not statistically significant (Fig. 2, Panel e and g). Rates of malaria cases in pregnancy seen in health facilities did not show statistically significant changes. Caesarean section rates, for which the Dori–Djibo pair of districts had to be excluded since the service was not offered there, also did not show much difference between intervention and control districts, a finding which was also statistically insignificant (Table 2). Also, intra-facility maternal mortality ratio (MMR) was reduced more in the intervention districts compared to the control districts. However, this outcome was statistically insignificant (Table 2).

**Fig. 3:**
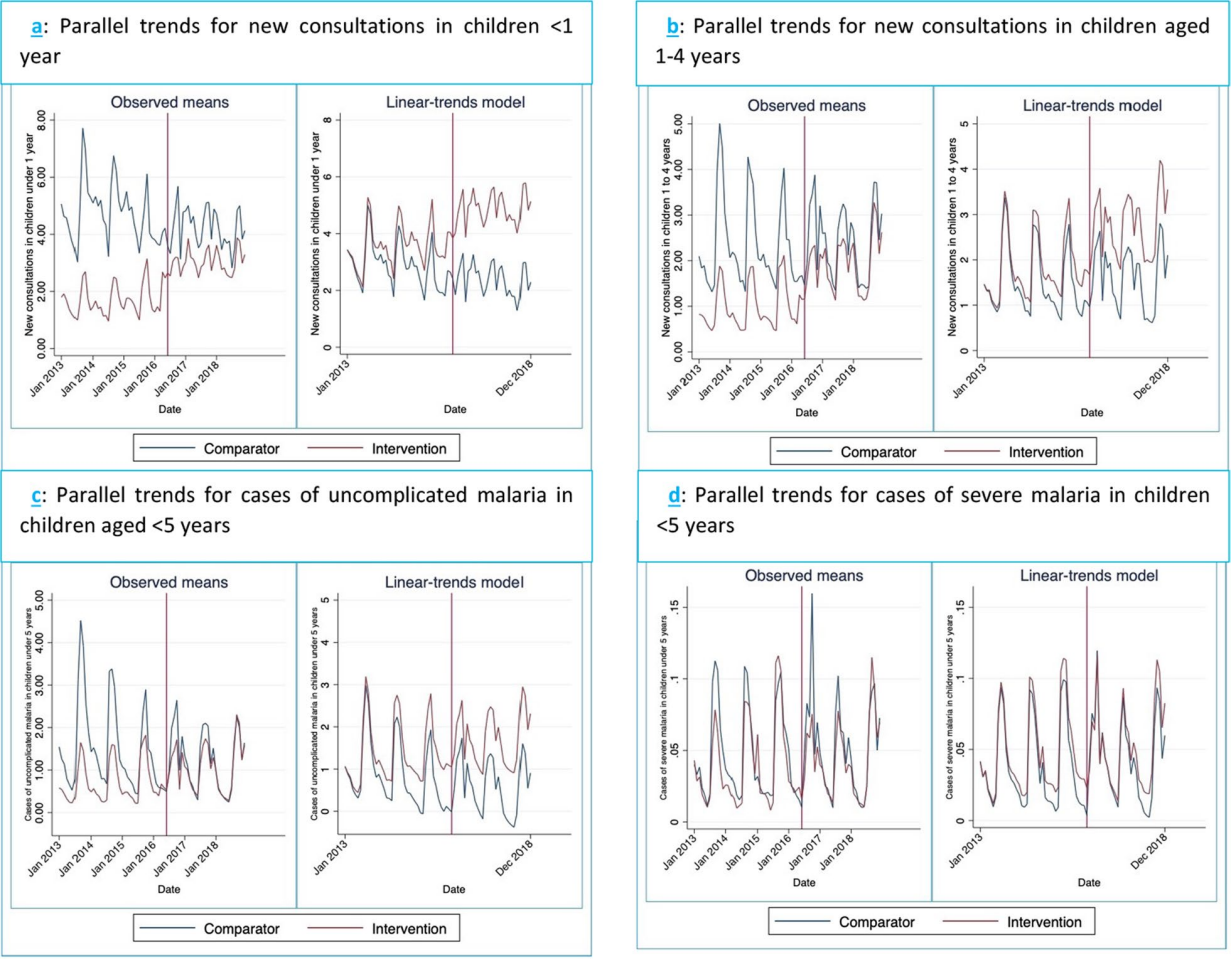
Graphical diagnostics of parallel trends for child health indicators

Figure 3; Panel a–d graphically depicts the modelled parallel trends for the same group provided in Table 2. The intervention led to an increase in rates of new consultations in children under 1 year (Coeff 1.80; 95% CI 1.13–2.47, *p* < 0.001) (Fig. 3; Panel a) and an increase in rates of new consultations in children 1–4 years (Coeff 0.81; 95% CI 0.50–1.13, *p* = 0.001) (Fig. 3; Panel b) in the intervention districts compared to the comparison districts and above what would have been observed in the intervention districts in the absence of the intervention. Further analysis supported the existence of a parallel trend between the intervention and comparison districts before the intervention, which further strengthens the causal effect of the intervention on new consultations in children less than 1 year and 1–4 years. The policy also led to an increase in the rates of uncomplicated malaria cases in children under 5 years seen in health facilities in the intervention districts (Coeff 0.59; 95% CI 0.44–0.73, *p* < 0.001) compared to the comparison districts and above what the trend would have been in the intervention districts in the absence of the intervention (Fig. 3; Panel c). However, there was not enough evidence to prove that a parallel trend existed between intervention and comparison districts prior to the intervention (*p* = 0.002). Other child health indicators assessed did not show significant differences. Rates of severe malaria cases in children under 5 years seen in health facilities did not show any statistically significant changes in the analysis (Table 2 and Fig. 3; Panel d). Also, the intra-facility early neonatal mortality rate was slightly reduced in intervention districts compared to control districts, although this outcome was not statistically significant (Table 2).

## Discussion

In this study, we set out to assess the effect of the Gratuité policy on the utilization and outcomes of MNCH services comparing 42 months before and 30 months after its implementation in conflict-affected areas of Burkina Faso. As per our results, though the rate of health facility delivery was found to have increased in intervention areas compared to control areas, this was not statistically significant. It is entirely plausible that the conflict may explain this modest non-significant increase in the rate of health facility delivery. Indeed, a previous study reported that a 3.8% reduction in the rate of health facility delivery a month following a terrorist attack in Burkina Faso [25]. However, there might be another explanation. A previous interrupted time-series analysis (ITSA) study which assessed impact of SONU and the NGO-led pilot schemes in some of the districts that we also studied showed that there was an average 4% annual increase in facility-based delivery which plateaued after 3 and 4 years of SONU and the NGO-led pilot schemes respectively. This trend, observed between 2008 and 2016, was just before the launch of the Gratuité policy^.^[11]. It is possible that the timing of the Gratuité policy launch might explain why our findings showed some increase in delivery rates which was not statistically significant. The question is, “Has the utilization rate for childbirth fully plateaued for mothers”? According to the Demographic Health Survey, the proportion of deliveries in a health facility increased from 38% in 2003 to 94% in 2021. Over the same period, the change in proportions was greatest in rural areas, where a rate change from 31% in 2003 to 93% in 2021 was observed [26]. Across the country, the increasing trend precedes the launch of the Gratuité policy in 2016. Situating this evidence in the context of our findings, it appears while the user fee reduction scheme (SONU) led to some of the increments seen since its launch, the user fee exemption scheme may not have led to any additional significant gains in utilization rates keeping in mind that an almost ceiling utilization rates had already been achieved.

For ANC, there was no statistically significant positive upward trend as was expected for ANC1 rates, ANC5+ rates, rates of women seen in the first trimester of pregnancy, and rates of malaria cases in pregnancy seen in the health facility during ANC. As with health facility delivery, the conflict may explain this finding, especially as there is strong evidence that conflict affects ANC utilization in fragile and conflict-affected settings [27]. With employment opportunities limited in such settings, pregnant women may be challenged to secure the funds needed to pay for the service and transportation fees associated with ANC. However, the Gratuité policy was designed to eliminate the user fee. As such, it is surprising that we have not found a significant difference. In a non-conflict setting of Malawi, a similar user fee exemption policy led to a significant 15% increase in ANC1 after the introduction of the policy [28]. In Burkina Faso, the national bulletin called *Annuaire Statistique* published in 2019 did not suggest that there have been any major changes in ANC utilization from 2015 to 2018, with annual utilization rates of ANC1 visits ranging from about 80 to 82% [29]. As this is consistent with findings from our study, the reason for this marginal increase in ANC1 rates needs to be further explored with the women themselves.

For PNC received by women, we found that the policy led to a significant increase in rates of 6th day postnatal visits. However, though rates of 6th hour and 6th week postnatal visits were found to have increased in intervention districts compared to control districts, these were not statistically significant. There is a potential explanation for the varied findings for the 6th hour, 6th day, and 6th week. For the 6th hour PNC, this care takes place immediately after delivery, so this makes sense since we also found an insignificant increase in delivery care. The significant increase in rates of 6th day postnatal visits because of the Gratuité policy is an important finding, more so in the context of conflict, as data from the National Demographic Health Survey published in 2012 shows that as many as a third of women who delivered in health facilities in the regions of the districts included in this evaluation had not received PNC by the 6th day [21]. Lower utilization rates were reported in Séguénéga, one of the health districts we studied, where over 75% of women did not return to health facilities for the 6th day postnatal visit in 2006. The main reasons given by mothers for this lack of use were lack of information, forgetfulness, and unwillingness [30]. Indeed, the significant increase in rates of the 6th day postnatal visit may simply be driven by increased demand for the service since women do not have to pay for care. However, this is unlikely since other maternal health services that we assessed did not point to similar strong association. Another potential and possibly more plausible reason may be because of their children. This 6th day PNC visit is linked with immunization visit for the child. Anecdotal evidence from NGOs responsible for policy verification suggests that there is a community moral pressure on women to actually make sure that they take their newborns to health facilities to benefit from the free care that the Gratuité policy offers because if the child were to die because of a vaccine-preventable disease, the women would be blamed for it. This argument is made stronger by the non-significant increase in the rate of 6th week postnatal visit that we observed, since it does not coincide with the expected weeks for facility visits that mothers will make for their children in line with the national vaccination schedule [31].

For the children, our study showed significant increases in new consultations in children under 1-year, new consultations in children 1–4 years, and rates of uncomplicated malaria cases seen in children under 5 years. Again, in the context of conflict, this is an important finding, as the Demographic and Health Survey showed that in 2010 only half of the children with one of either malaria, pneumonia or diarrhea were treated in health facilities [21]. In a recently published nationwide single arm interrupted time series on the Gratuité policy which had no comparison group, authors reported a 57% (incidence rate ratio (IRR) = 1.57; 95% CI 1.2–2.0) increase in rate of health facility visits in the month immediately following the policy’s launch [32]. A previous ITSA study in Burkina Faso, which assessed the effect of a previous user fee exemption policy combined with quality improvement interventions in 2008, showed that the policy more than doubled the utilization rate of health services for children under 5 years in the immediate period following its launch. However, after this initial period, the pace of growth slowed and subsequently stabilized by 3 years and 7 months before decelerating slowly towards the sixth-year post-implementation [33]. A previous publication suggested a significant 14.8% increase in cases of fever nationally comparing before and after the launch of the Gratuité policy [34]. A similar fee exemption policy experiment implemented in the conflict-affected Sahel region of Burkina Faso before the Gratuité policy led to a doubling of health facility attendance for children after adjusting for the size of the health facility, districts, secular trend, and seasonal variation [35]. Another study in Mali showed that removing user fees for vulnerable groups trebled utilization of health services for malaria treatment amongst children [36]. Our study specifically found that rates of service utilization for uncomplicated malaria cases and new consultations significantly increased. This aligns with findings from a qualitative study with multiple stakeholders of the policy in Burkina Faso in which health workers reported that they observe earlier presentation of children in facilities since the implementation of the policy [37]. However, we did not see a significant increase in children with complicated cases presenting in health facilities. This finding suggests that even in the context of conflict, the Gratuité policy motivates parents to be more proactive in seeking care for uncomplicated cases, which could morph into complicated cases if delayed, for their children.

Regarding care outcomes, there was more reduction in post-intervention intra-facility MMR and early neonatal mortality rates comparing the intervention districts to the control districts, though these outcomes were found to be statistically insignificant. This was the policy intention at its inception [14], however, as the findings were not statistically significant, it is not possible to draw any conclusion.

### Strengths and limitations

There are some strengths worth highlighting as regards this evaluation. First, we focused on conflict-affected districts bearing in mind that available evidence had not captured the effect of insecurity ﻿on the quantity and quality of healthcare services provided through the Gratuité policy [32]. Second, despite the almost immediate national scale-up of the policy, which had limited robust two-arm impact evaluations of the scheme to date [32], we were able to identify both intervention and comparator districts with clear divergent characteristics for our study. Third, we selected districts that had not been exposed to the PBF scheme, which was also implemented in the country and had been shown to increase service utilization for mothers in intervention areas [22]. By excluding these areas, we were able to demonstrate impact related to the Gratuité policy alone. Fourth, the DID approach used in our study allowed control for changes in data processing and reporting practices which could explain the trends in service utilization in pre- and post-policy periods. Fifth, our study assessed the full MNCH continuum of care allowing us to understand interactions between the services.

However, there are limitations to the evaluation. First, it is possible that findings from our study cannot be generalized to other areas of conflict in the country since we could only focus on eight health districts within the four regions that are most affected in Burkina Faso. However, the health districts included in our study are situated in some of the worst-performing regions in the country [21]. As such, our results can be interpreted as a base case representation of the impact of the Gratuité policy on service utilization and outcomes. Second, we only had annual data for metrics used as denominators in this evaluation (e.g., number of expected pregnancies, number of live births, number of women of childbearing age, the total population of children < 1 year etc.). As such, monthly statistics were only obtained by dividing annual data by 12. Third, we could not compare the full period of the Gratuité policy implementation, as data was missing for the year 2019. In any case, the Coronavirus Disease 2019 pandemic, which has been shown to have affected MNCH service utilization, started in 2020 [38–40]. Indeed, our results could be different if we could continue till now, especially within the context of insecurity currently ravaging some of the districts that we selected for our study [41].

### Implications for policy, practice, and research

As per evidence from this evaluation, there is a clear case for sustaining the Gratuité policy even in conflict-affected areas, especially as user fees are a huge barrier to service utilization in LMICs, even outside conflict settings [42, 43]. At the very least, the Gratuité policy allows parents to be more proactive about seeking care for their children, even in areas with security challenges. This is more so important as evidence from Burkina Faso has already shown that perceived lack of safety in an area where a health facility is situated significantly reduces seeking of appropriate care for childhood fever to such facility even when a PBF scheme is being implemented [44]. It is well recognized that presentation in health facilities with uncomplicated malaria ensures minimal progression to complicated cases and ultimately minimizes poor outcomes, including mortality amongst children under 5 years, which we have not reported in this evaluation. Indeed, the Gratuité policy remains a pragmatic approach and has demonstrated effectiveness. Currently, the Gratuité policy is funded from general government revenues. Gratuité is budgeted as a specific activity within Burkina Faso’s program-based budgeting approach, and since 2017, there has been consensus on “ensuring the implementation of the Gratuité policy”. The simplest solution to the challenge of funding Gratuité will be to ensure the execution of this budget line. However, the Government also needs to fund several competing priorities, more so in a period of insecurity. The government should implement hypothecated or ring-fenced taxes for health. These have been implemented in other African countries to varying effectiveness in raising funds [45], and this could be an option for funding the Gratuité policy.

In addition, community mobilization programs might be needed, as when findings from our study are combined with those from other robust impact evaluation studies conducted in Burkina Faso and conclusions from the recent DHS, it suggests that some policy gains could have reached a ceiling. In this instance, grassroots community-led interventions may be needed to ‘move the needle further’ towards realizing UHC. The recent mobilization of Agents de Santé à Base Communautaire (ASBC), who are volunteer community health workers, offers an opportunity for improving MNHC service utilization in conflict-affected areas. This scheme should be sustained while ensuring that incentives and adequate support are put in place to maximize effectiveness of that ASBCs can bring care closer to women and children in affected communities [46, 47]. Finally, effective innovations such as the Missed Opportunities for Maternal and Infant Health project implemented in Burkina Faso, which integrated PNC in infant immunization services, should be considered for mainstreaming into routine care practice [48]. Integrating services will enable mothers (and their children) who rarely visit facilities to benefit from a range of related services.

For research, there is a need to further explore the reason underpinning non-significant observation of some MNCH services (ANC and delivery) in conflict-affected areas. In addition, there is value in considering the conduct of a realist evaluation which will allow the exploration of potential causal explanations of findings observed following the implementation of the policy. The realist evaluation not only assesses whether an intervention worked or not, but realist evaluators also interrogate “What works (or does not work)? For whom (and to what extent)? In which circumstances does it work? How and why does it work?” [49–51]. Future research should also explore utilization and outcomes from an equity lens to understand if particularly vulnerable populations have benefited from the policy.

## Conclusion

This study showed that, for the most part, the Gratuité policy is achieving what it set out to do, which is to increase access to care by removing financial barriers. The policy contributed to a significant increase in rates of 6th day postnatal visits for women and significant increases in new consultations for children < 1-year, new consultations in children 1–4 years, and consultations for uncomplicated malaria in children < 5 years. Further studies are needed to establish if the policy significantly improves care outcomes. As the country moves towards the goal of realizing UHC, sustained investment in the Gratuité policy is warranted, even in conflict-affected areas [52].

## Data Availability

Data used in this study is available from the Ministry of Health and Public Hygiene on reasonable request.

## Abbreviations

ASBC: Agents de Santé à Base Communautaire
ANC: Antenatal care
CI: Confidence interval
CMA: Centre médical avec antenne chirurgicale
CSPS: Centre de santé et de promotion sociale
DHS: Demographic and Health Survey
ECHO: European Civil Protection and Humanitarian Aid Operations
HMIS: Health Management Information System
LMICs: Low-and Middle-Income Countries
MOH: Ministry of Health
MNCH: Maternal, newborn, and child health
MMR: Maternal mortality ratio
SDGs: Sustainable Development Goals
SONU: Soins Obstétricaux et Néonataux d'Urgence
NGO: Nongovernmental organization
PNC: Postnatal care
SONU: Emergency Obstetric and Newborn Care
UHC: Universal Health Coverage
